# Exploring the long-term effect of plastic on compost microbiome

**DOI:** 10.1371/journal.pone.0214376

**Published:** 2019-03-25

**Authors:** Ebenezer Oluwaseun Esan, Lord Abbey, Svetlana Yurgel

**Affiliations:** Department of Plant, Food, and Environmental Sciences, Dalhousie University, Truro, NS, Canada; Justus-Liebig-University, GERMANY

## Abstract

Little is known about the ecology of microbial plastic degradation. In this study, we employed next generation amplicon sequencing to assess the effect of low-density polyethylene (LDPE) films on the structure of bacterial and fungal communities in four mature compost piles with age ranging between 2 and 10 years. While, bacterial *Proteobacteria*, *Bacteroidetes*, *Actinobacteria* and fungi *Ascomycota* were most abundant across all facilities, our data indicated significant differences in compost microbiomes between compost facilities, which might be related to compost chemical parameters, age of piles and characteristics of the feedstock. In addition, a substantial shift in the interaction pattern within microbial communities from bulk and plastic-associated (PA) compost was detected. For example, cooperation between *Firmicutes Bacillaceae* and *Thermoactinomycetaceae* was detected only in PA compost. However, based on the analysis of the diversity indices and the relative abundances of microbial taxa we can conclude that the presence of plastics in compost had no significant effect on the structure of microbial community.

## Introduction

Plastics have a wide range of application in virtually all aspects of human life in both domestic and commercial settings. The current rate of increase in global use of polyethylene and plastic products is approximately 12% per annum, and this continues to rise [1]. For instance, consumer demand drove global production to approximately 140 million tons of synthetic polymers, which increased by 1.74-fold over the past 15 years to about 243 million tons [2]. As a result, the amount of global plastic wastes have tremendously increased, posing various degrees of threat to the environment, ecosystems, economies and all life forms. Plastic contamination of compost, an organic soil amendment used in agriculture to enhance soil health and productivity, may have a wide range of negative impacts on the environment and agro-ecological systems. These concerns have been expressed by the public including environmental advocates, growers and researchers [3]. For instance, plastics in compost can cause major environmental threats due to their inability to breakdown or their low rate of breakdown which thereby, may lead to environmental pollution, blockage of water ways and death of marine and fresh water flora and fauna [4].

Plastics can negatively affect the soil ecosystem by releasing toxic substances and inhibiting soil dwelling [5]. Furthermore, plastic-associated toxic compounds can potentially enter the food chain and affect human health [6]. Another possibility is adverse effects of plastic contamination on seed germination, root penetration, nutrient and water flow, and root uptake of water and nutrients from soil [7]. Although another study suggested no effect of plastic contamination on seed germination [8]. Additionally, essential resources such as water and soil nutrients are not accessible to plants due to impermeability of these pieces of plastics.

Composting is an environmentally friendly and organic method of waste management. Historically, composting has been used to recycle agricultural wastes and return the composted organic matter into the soil to maintain soil fertility and crop productivity with minimum application of synthetic chemical fertilizers [9]. During composting, the diversity and structure of microbial communities, as well as chemical and physical properties of the composting substrates change dramatically in the course of several weeks [10–13].

One of the main problems facing the compost industry is contamination from plastic wastes. In some cases, visible plastic wastes in compost can reach up to 1.2 g per kg of compost [4, 14]. Low-density polyethylene (LDPE) is an example of thermo-plastic comprised of long chain unlinked polymer molecules and used to make thin, flexible plastics like wrapping films, grocery bags, sandwich bags and a variety of soft packaging materials. Due to the inert nature of plastics such as LDPE, their decomposition requires a prolonged period (up to 1000 years) that goes far beyond the normal period for the various biotic and abiotic steps in the composting process, which can last from several week to several month depending on the type of composting method. Biotic plastic degradation is a natural process by which microorganisms breakdown plastics into smaller units in order to use the carbon sources for energy and growth [15, 16]. Studies on biodegradation of several types of plastics in soils revealed that some fungi and bacteria species are capable of degrading plastics into carbon and energy for cellular metabolism [15, 17]. However, little is known about the ecology of microbial plastic degradation and the effect of plastic contamination on compost microbiome.

In this study we employ next-generation amplicon sequencing to better understand the ecological effect of plastic contamination of compost by assessing LDPE plastic-influenced species sorting effect on bacterial and fungal communities in mature compost piles at the age range of 2 to 10 years. We analyze diversity and structure of microbial communities associated with both mature bulk compost and compost associated with partially degraded plastic from each of four major compost facilities across Nova Scotia (Canada). We also applied microbial co-occurrence analysis to evaluate the effect of plastic on the interaction within microbial communities and to identify niche specific modules, which could provide the insight on the function of the microbiome in plastic-influenced environments. We hypothesized that plastic environments may cause a shift in microbial cooperation improving microbiome adaptation and efficiency of carbon utilization in plastic-influenced environments.

## Materials and methods

The study was carried out on private land and the owners of the land gave permission to conduct the study on these sites, and therefore no specific permissions were required for these locations/activities

### Sample collection and processing

Samples of compost and plastics were collected in August and September of 2016 from four different composting facilities in Nova Scotia, Canada (Table 1). Five samples of 5 g of partially decomposed LDPE films (parts of plastic bags introduced into the compost with household and grocery stores waste) were randomly collected from compost piles in each location. In addition, 500 g of bulk compost within 10-cm radius around the sampled plastics were collected at each location using a sterile hand auger. The plastic and compost samples were kept in sterile plastic bags and immediately placed in a cooling box with icepacks before transporting them to the laboratory. The samples were then processed within 24 hrs.

**Table 1:** Description of compost facilities, age of compost and source of feedstock.

Name of facility	Coordinate	Age of pile, years	Source of feedstock^a^	Bulking material
Colchester Composting Facility, Kemptown	45°27'24.6"N 63°06'20.1"W	2	Colchester	Wood chips during the winter season
Fundy, Fundy Compost Inc., Brookfield	45°15'01.5"N 63°20'46.9"W	10	Halifax, East Hants regional municipality	Wood shelving/woodchips and woods
Valley-Northridge Farms, Aylesford	45°03'20.9"N 64°50'27.6"W	2	Kings, Queens and Annapolis counties	Hays and straw during the winter season
Guysborough Composting Facility, Boylston	45°29'33.7"N 61°32'15.2"W	3	Antigonish, Port Hawkesbury, and Guysborough counties	Hays and straw during the winter season

^a^ The feedstock include household waste, yard waste, and unsorted food waste mainly from residential and grocery stores

#### Bulk compost processing

Approximately, 10 g of the bulk compost samples were sieved using 2-mm sieve and kept at -80°C for DNA isolation. DNA was isolated from 0.25 g of compost. The rest of the bulk compost samples were sieved using a 5-mm sieve before storing at -20°C for the analysis of compost chemical parameters.

#### Plastic-associated (PA) compost processing

Partially decomposed LDPE film samples were individually placed into conical flasks with 150 ml of sterile 10% glycerol and shook for 15 min, followed by sonication for 15 min. The films were removed and placed into clean flasks. The entire process was repeated by following the same steps described above. The solutions from the two cleaning steps were combined and centrifuged at 3,000 x g rpm for 30 min. The supernatants were then decanted and the pellets were transferred into 1.5 ml Eppendorf tubes and centrifuged again at 4,000 x g rpm for 10 min. The supernatant was discarded, and the samples were stored at -80°C until processing for DNA isolation. DNA was isolated from 0.25 g (wet weight) of PA compost.

### Compost chemical parameters

The analysis was done at the Nova Scotia Department of Agriculture & Food Operations Laboratory Services (Harlow Institute). Each compost sample was characterized by determining pH, total nitrogen (N, %), organic matter (OM; %), phosphate (P_2_O_5_; kg/ha), potash (K_2_O; kg/ha), calcium (Ca; kg/ha), magnesium (Mg; kg/ha), sodium (Na; kg/ha), sulfur (S; kg/ha), iron (Fe; ppm), manganese (Mn; ppm), copper (Cu; ppm), zinc (Zn; ppm), aluminum (Al; ppm), and cation-exchange capacity (CEC;meq/100 g) according to standard procedures [18, 19].

### DNA extraction and sequencing

DNA was extracted using the PowerSoil DNA Isolation Kit (MO BIO Laboratories, Carlsbad, CA, USA) according to the manufacturer’s protocol. DNA quality and concentration were measured using a NanoDrop 1000 spectrophotometer (Thermo Scientific, Waltham, MA, USA). At least 50 ng (10 μL) of DNA sample were sent to the Centre for Comparative Genomics and Evolutionary Bioinformatics Integrated Microbiome Resource, CGEB-IMR (http://cgeb-imr.ca/), at Dalhousie University for V6-V8 16S rRNA gene (16S) and fungal internal transcribed spacer (ITS)2 region (ITS) library preparation and sequencing. Samples were multiplexed using a dual-indexing approach and sequenced using an Illumina MiSeq with MiSeq Reagent kit v3 (2 x 300 bp). All PCR procedures, primers, and Illumina sequencing details were as described in [20] and [21].

### Sequencing data processing

Sequence data processing and OTU picking were described in our earlier work [21]. Briefly, we used the Microbiome Helper standard operating procedure to process and analyze the sequencing data [22]. Overlapping paired-end reads were stitched together using PEAR (v0.9.6) [23]. We then ran FASTX-Toolkit (v0.0.14) [24] and BBMap (v35.85) [25] to filter out low quality reads. Finally, we ran USEARCH (v6.1) [25, 26] to screen out chimeric reads.

### Operational taxonomic unit (OTU) picking and statistical analyses

After filtering, we ran open-reference OTU picking using QIIME wrapper scripts [27]. Specifically, SortMeRNA (v2.0-dev) [28] was used for the reference OTU picking steps (with sortmerna_coverage = 0.8) and sumaclust (v1.0.00) [29] for the de novo OTU picking steps (with 10% of the failures subsampled). OTUs that contained fewer than 0.1% of the total sequences were removed. Alpha-diversity (Chao1 richness, Simpson evenness and Shannon diversity) and beta-diversity (Bray-Curtis ecological distances) [30] metrics were generated using QIIME. Variations in sample groupings explained by Bray-Curtis beta-diversity distances (Adonis tests, 999 permutations) were run in QIIME to calculate how sample groupings are related to microbial community structure. Correlations between community structure (Bray-Curtis distances) and soil factors dissimilarity matrices (Mantel test) were analyzed using QIIME function compare_distance_matrices.py. Spearman and Tukey’s pairwise tests were run using Minitab v. 18.1 software (Minitab Inc., PA, USA). Analysis of taxonomic profiles was performed using the STAMP software package [31]. Corrected P-values (q-values) were calculated based on Benjamini–Hochberg FDR multiple test correction. The sequences generated in this study are available in the NCBI sequence read archive under the BioProject accession ID PRJNA485067.

### Co-occurrence network construction and analysis

The co-occurrence analysis was performed using the CCREPE (Compositionality Corrected by REnormalization and PErmutation) R package [32] as it was described previously [33]. Microbes were grouped at the genus level. The taxa found in less than 5% of samples were removed from the analysis. The taxa represented by less than 1% of the reads in all samples were also removed. First, the co-occurrence and co-exclusion patterns in the samples were scored. The resulted were filtered to remove non-statistically significant relationships. The associations with correlation coefficient (ns-score) > 0.5 or < -0.5 and a p-values of < 0.01 compared to 1000 bootstrapped permutations were considered significant and included as network edges weighted by their correlation coefficient. We generated networks based on correlations with p-values of < 0.01 and < 0.0001. The networks were analyzed and visualized with Cytoscape [34] and were represented as graphs with microbial groups as vertices/nodes and the edges as interaction types. An “edge-weighted spring embedded” layout in which positive correlations (blue) are pulling samples together forming clusters, while negative correlations (red) are pushing the samples apart was used for co-occurrence network visualization.

## Results

### Compost associated microbial communities

The structure of bacterial and fungal communities in the bulk and the PA compost from the four compost-processing facilities were analyzed. A total of 838,769 and of 825,446 high-quality 16S rRNA and ITS reads, respectively, were obtained from 40 samples comprising five bulk and five PA compost samples from each facility. The 16S and ITS dataset were normalized to a depth of 5,545 and 776 reads, respectively. These reads were distributed between 4,391 bacterial and 653 fungal OTUs at 97% identity, respectively. According to the taxonomic affiliation of the OTUs, bulk and PA compost hosted 593 bacterial and 198 fungal classes. The bacterial communities were dominated by *Proteobacteria* (42%), *Alphaproteobacteria (*17%), *Gammaproteobacteria* (16%), *Deltaproteobacteria* (5%), *Betaproteobacteria* (5%), *Bacteroidetes* (34%), *Actinobacteria* (8%), *Acidobacteria* (3%), *Gemmatimonadetes* (3%), *Firmicutes* (3%) and *Chloroflexi* (2%) (S1 Fig). The fungal communities were dominated by *Ascomycota* (66%), *Sordariomycetes* (23%), *Pezizomycetes* (13%), *Dothideomycetes* (5%), *Eurotiomycetes* (5%), *Leotiomycetes* (4%), *Basidiomycota* (25%), *Agaricomycetes* (22%), and *Zygomycota Mortierellomycotina* (7%). Unclassified fungi and *Ascomycota* were represented by 11% and 5% of all high-quality ITS reads.

**Fig 1:**
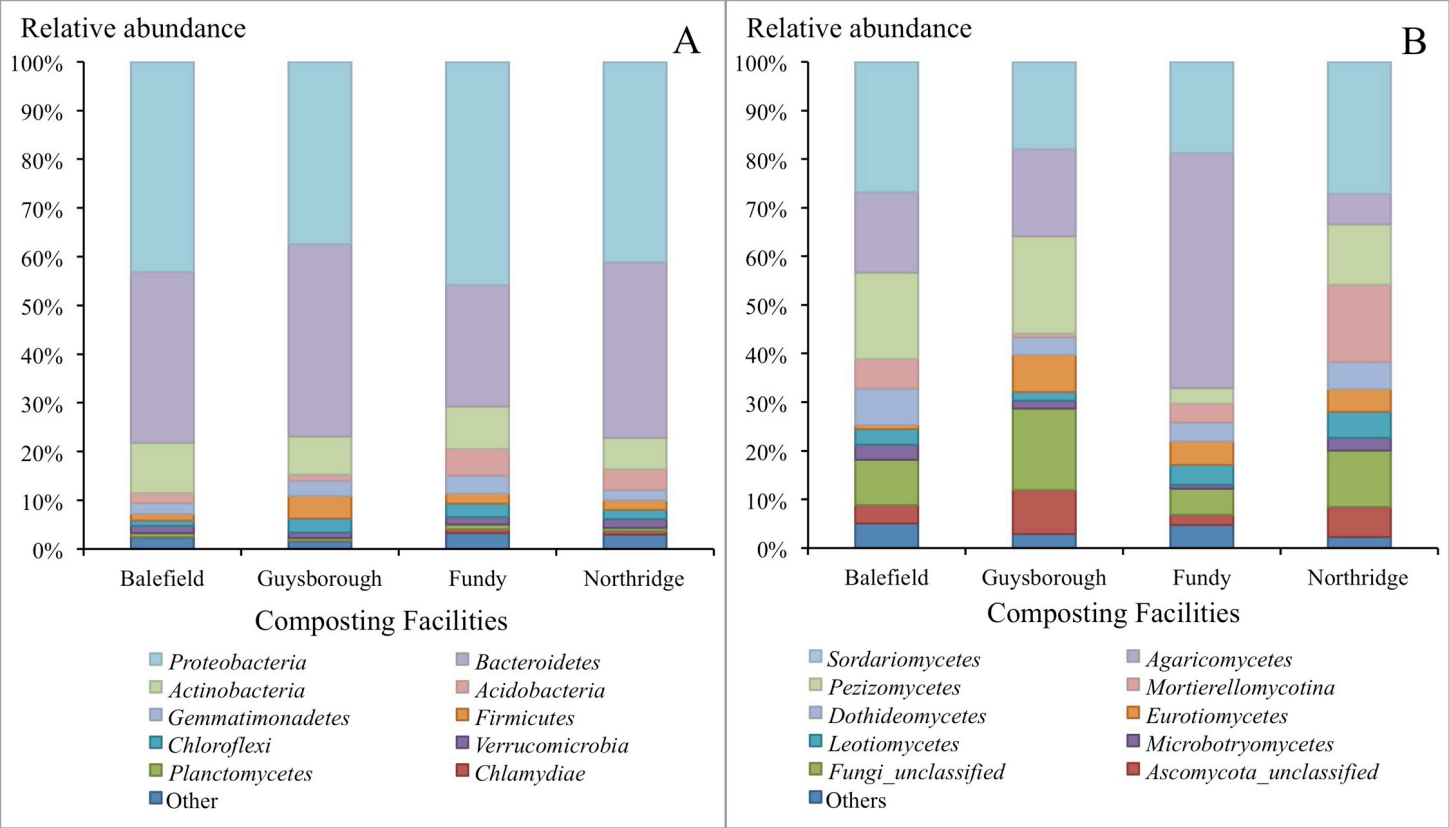
Relative abundances of microbial taxa present in the microbial communities from different composting facilities. Each bar represents microbial communities from both mature bulk and PA compost. (A)–Bacteria, 16S rRNA gene; and (B)–fungi, ITS.

**Fig 2:**
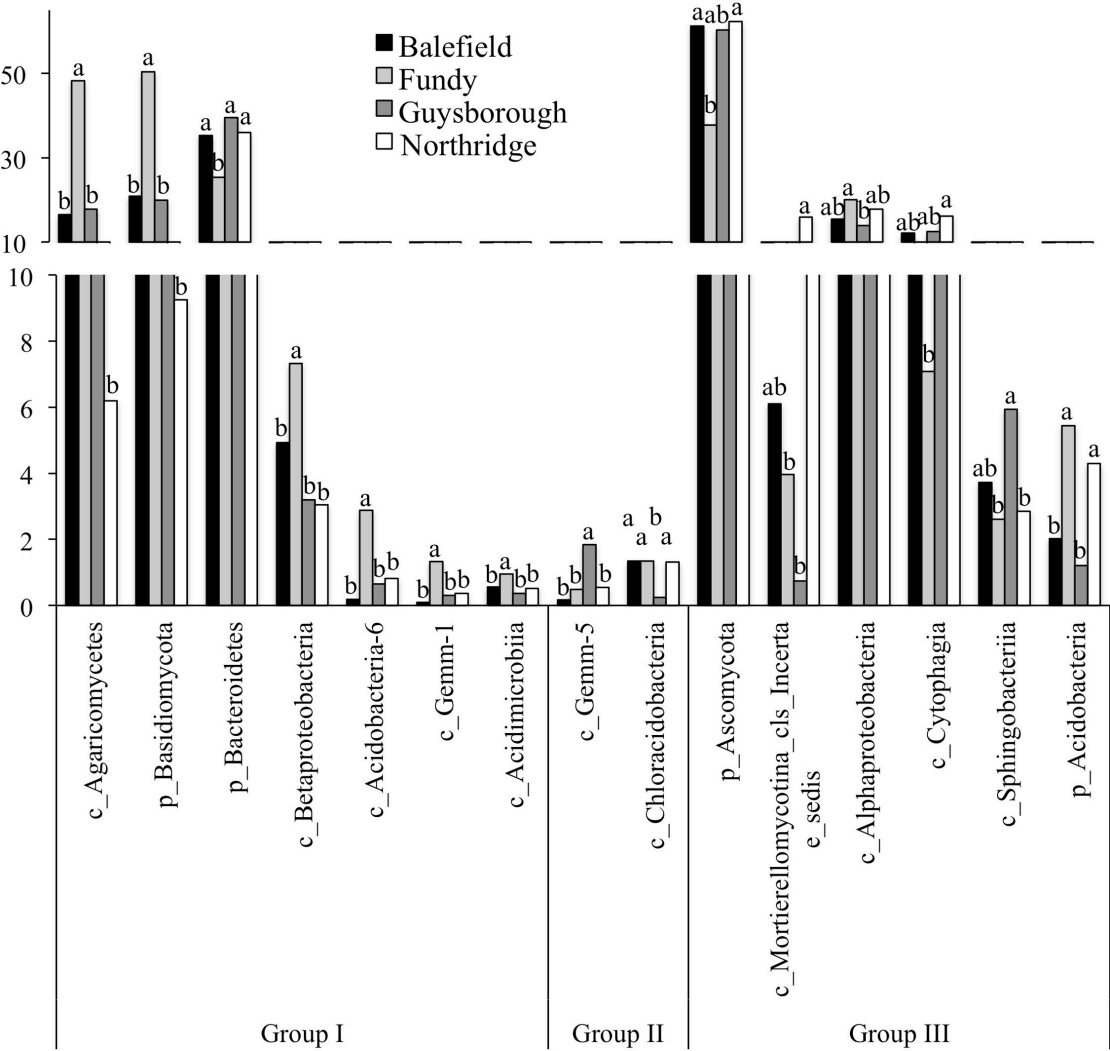
Microbial taxa those were significantly overrepresented in comparison between compost facilities. Corrected P-values (q-values) were calculated based on Benjamini-Hochberg FDR multiple test correction. Features with (Welch's t-test) q value <0.01 were considered significant and were thus retained. Group I–Microbial taxa that were significantly overrepresented or underrepresented in Fundy facility compared with other facilities; only microbial taxa represented by >500 total reads. Group II–Microbial taxa that were significantly overrepresented or underrepresented in Guysborough facility compared with other facilities; only microbial taxa represented by >500 total reads were considered; Group III–Microbial taxa represented by more than 5% of total reads and differed in their relative abundances between facilities. For each variable, data followed by different letters are significantly different according to Tukey’s pairwise test (P < 0.05).

Highly specific distributions of the bacterial and fungal taxa were observed in compost samples from different facilities (Fig 1). For example, fungi *Agaricomycetes* and bacteria, *Betaproteobacteria*, *Acidobacteria-6*, *Gemm-1*, and *Acidimicrobiia*, were relatively more abundant while *Bacteroidetes* were relatively less abundant in the Fundy facility compared with other facilities (Fig 2, Group I). Additionally, *Gemm-5* were overrepresented and *Chloracidobacteria* were underrepresented in Guysborough facility compared with all other facilities (Fig 2, Group II). The other microbial taxa represented by more than 5% of total reads and differed in their relative abundances between facilities included fungi *Ascomycota* and *Mortierellomycotina* and bacteria *Alphaproteobacteria*, *Cytophagia*, *Sphingobacteria* and *Acidobacteria* (Fig 2, Group III).

### Diversity of microbial communities

We used Principal Coordinate Analysis (PCA) to visualize dissimilarity between bacterial and fungal communities, which provided an easy way to explore and compare large datasets representing environmental microbiomes. This analysis showed no obvious visual separation between microbial communities from different compost facilities (Facility factor) (S2A and S2B Fig), both bacterial and fungal community structures were significantly influenced by Facility factor (S1 Table). Additionally, the age of the pile (Age of Pile factor) was also a significant factor separating the structure of bacterial and fungal communities. The Facility had a stronger effect on microbial community compared with Age of Pile. For example, 26% and 10% of variations in the distances between bacterial communities was explained by Facility and Age of Pile factors, respectively. Similarly, ~28% of the variation in the distances between fungal communities was explained by Facility factor and only 9% of that was explained by Age of Pile factor.

**Fig 3:**
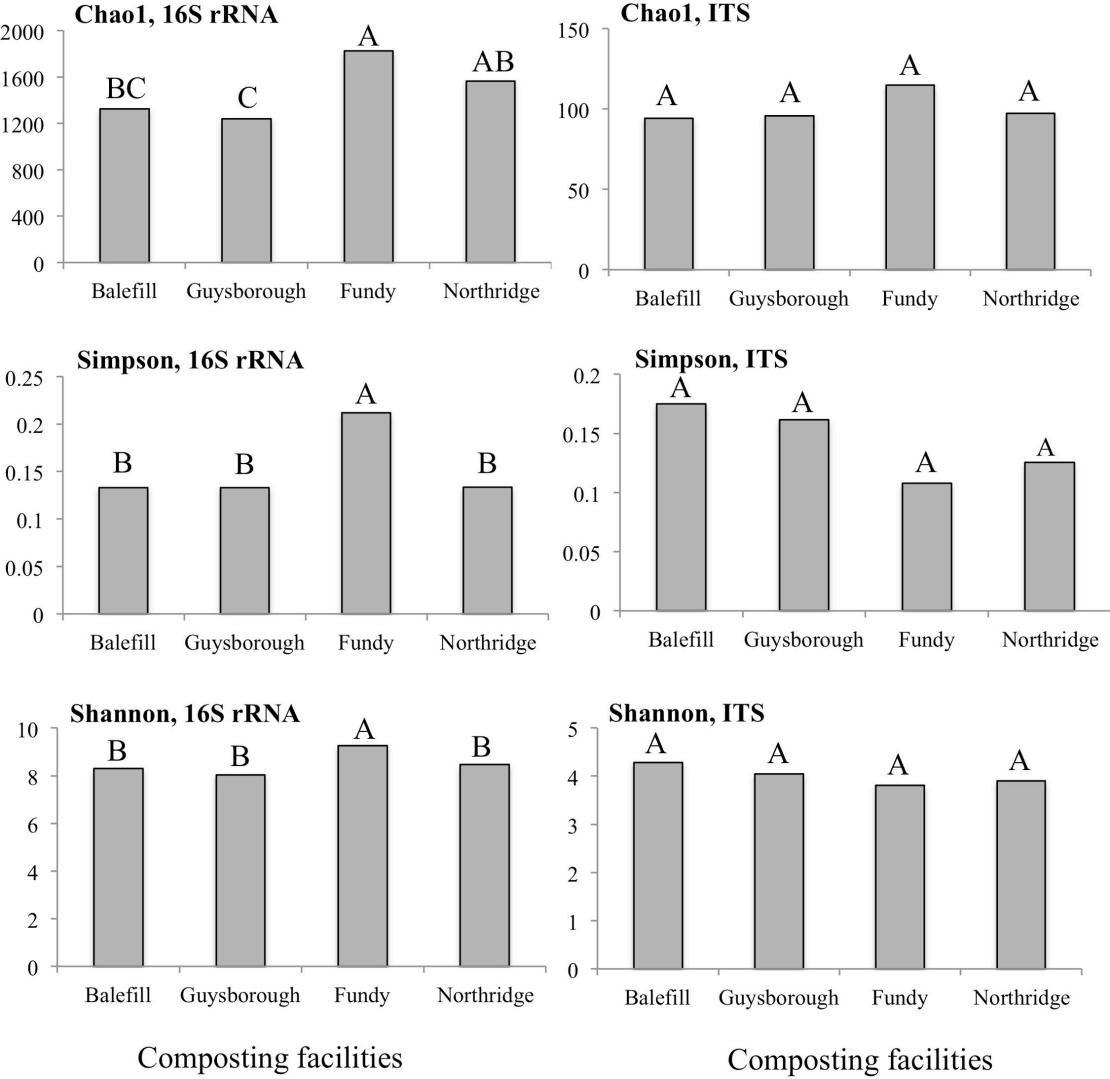
Estimated total species Chao1 richness, Simpson evenness and Shannon diversity. For each variable, data followed by different letters are significantly different according to Tukey’s pairwise test (P < 0.05).

Interestingly, pairwise comparisons of microbial alpha-diversity such as Chao1 richness, derived from the number of observation of species, Simpson evenness, indicating how equally abundant species in the community, and Shannon diversity, accounting for both abundance and evenness, revealed significant differences between Fundy and all the other compost facilities in bacterial alpha-diversity. Bacterial community from Fundy facility exhibited higher richness, evenness and diversity compared with the other facilities, while no significant differences in fungal alpha-diversity parameters were detected between all four facilities (S2 Table and Fig 3).

### Correlation between compost chemical parameters and the structure of microbial communities

**Fig 4:**
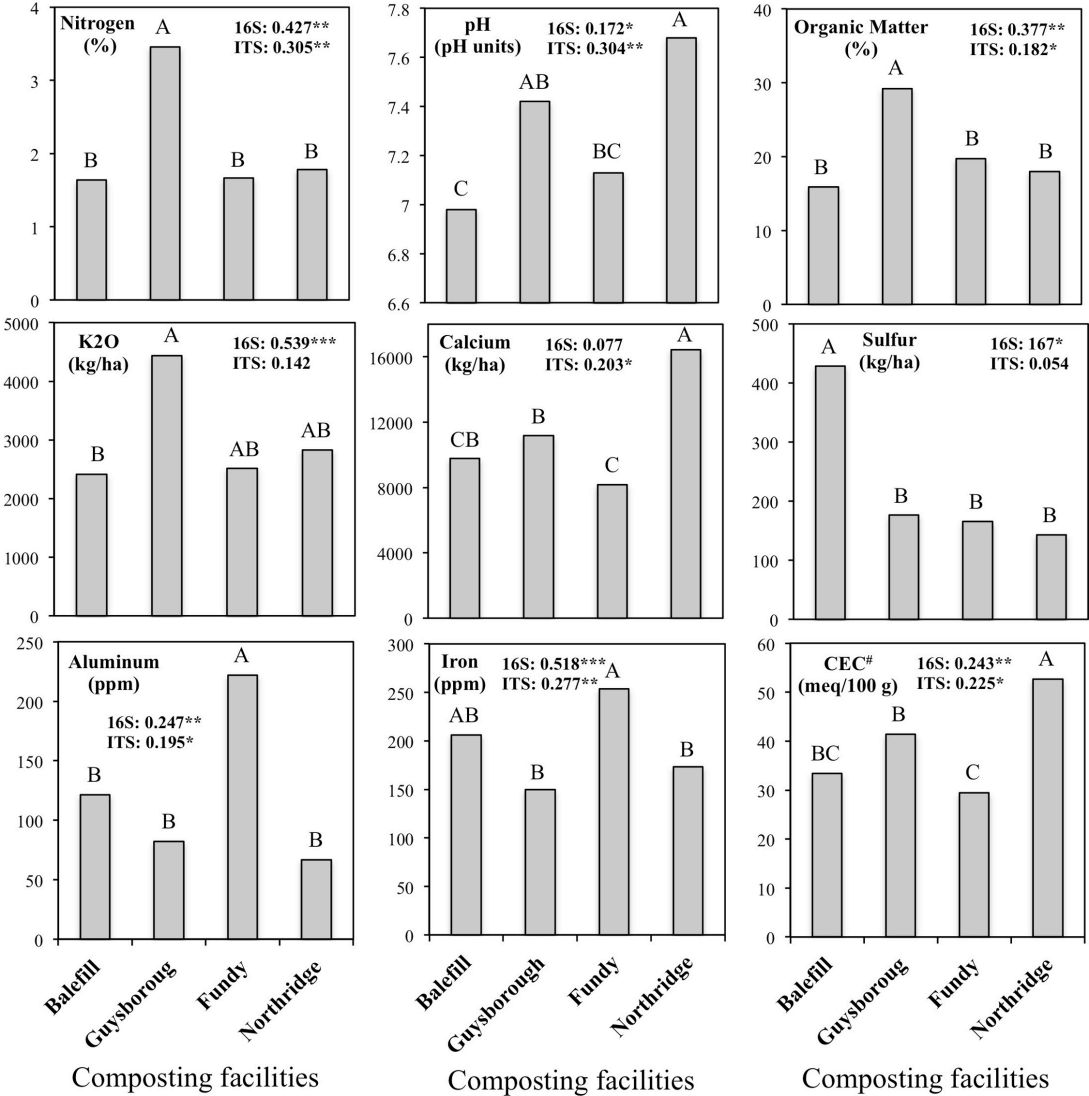
Mean soil chemical parameters and correlations between community structure and soil factors dissimilarity matrices. For each variable, data followed by different letters are significantly different according to Tukey’s test (P < 0.05). Adonis tests were used to assess whether beta-diversity is related to chemical soil factors, 999 permutations, R2, *p<0.05; **p<0.01, ***p<0.001. #–Cation-exchange capacity.

Compost chemical parameters differed between facilities. For example, bulk compost from Guysborough facility was the richest in nitrogen and organic matter (Fig 4 and S3 Table), compost from Balefill facility had highest sulfur and manganese content and compost from Fundy facility had highest aluminum content. The differences in compost nutrients composition might explain the variations in the microbial communities’ composition between the facilities. A significant correlation between community structure and several compost parameters was observed (Fig 4 and S4 Table). Compost nitrogen, pH, organic matter, aluminum, iron, and cation-exchange capacity were significantly correlated with both bacterial and fungal communities structure. Potassium, sulfur and sodium were correlated with variation in bacterial communities structure, while calcium affected fungal communities structure. Bacterial alpha-diversity was also correlated with some compost parameters. Bacterial community Shannon diversity was positively correlated with aluminum and iron and negatively correlated with potassium and calcium (S5 Table). On the other hand, we did not detect any correlation between compost parameters and Shannon diversity of fungal community.

### Effect of plastic on diversity and structure of microbial communities

PCA showed no visual separation between microbial communities from bulk and PA compost (Sample Type factor) (S2C and S2D Fig). Considering communities across all facilities, the analysis of strength and statistical significance of sample groupings (Adonis test) did not indicate that Sample Type influence bacterial or fungal community structures (S1 Table). Similarly, no statistical significance of sample groupings was detected when communities from each facility were considered separately. Likewise, no differences in bacterial or fungal alpha-diversity (S2 Table) or in the relative abundances of microbial taxa were detected between communities from bulk and PA compost.

### Microbial correlation in bulk and PA compost microbiomes

**Fig 5:**
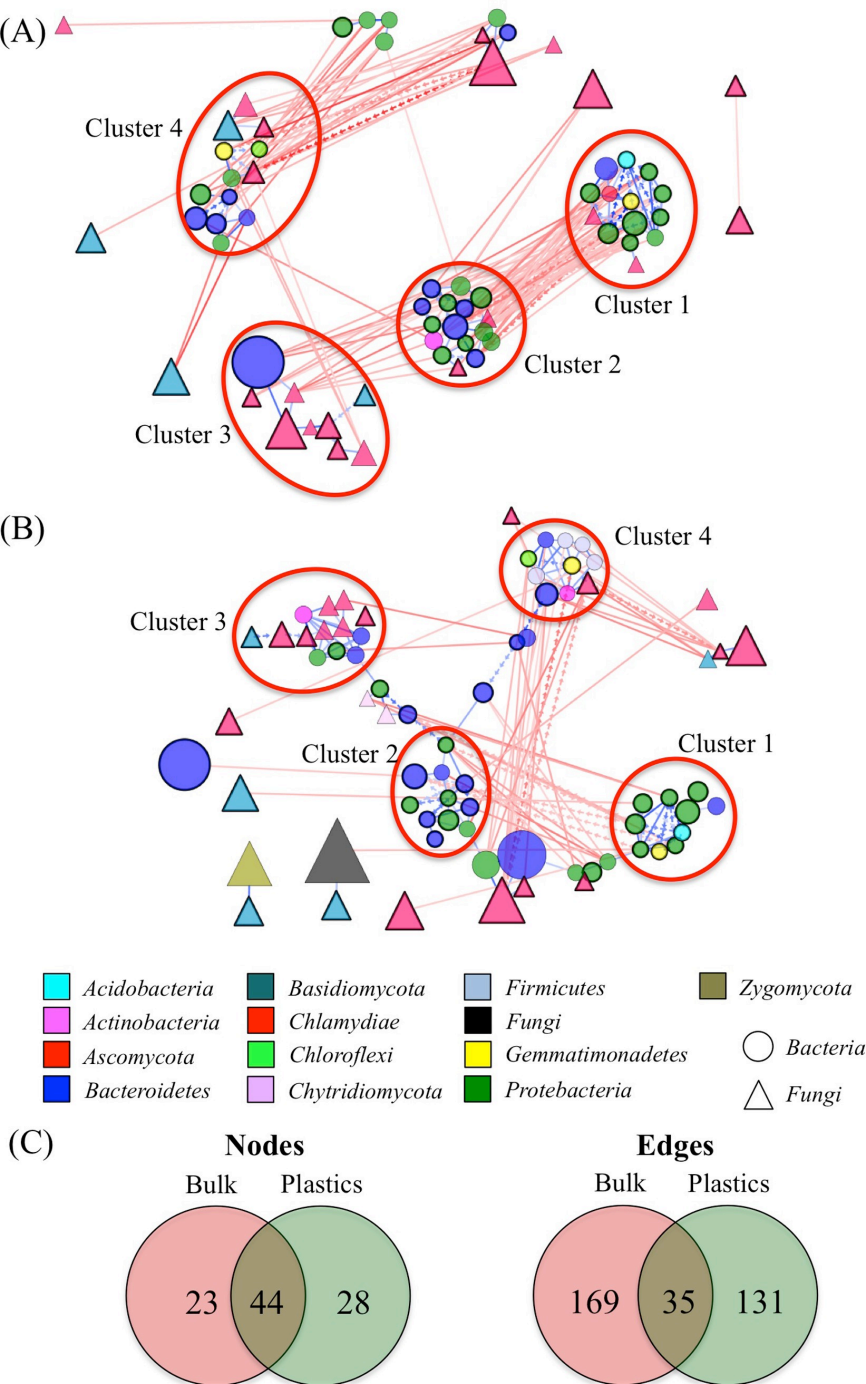
**Co-occurrence network generated by measuring abundance co-correlation between microbial taxa from bulk (A) and plastic-associated (B) compost.** Correlation base network analysis showing potential interactions between bacterial and fungal genera. The size of the node is proportional to a taxon’s average relative abundance across all the samples. The lines connecting nodes (edges/associations) represent positive (blue) or negative (red) co-occurrence relationship. The solid lines indicate niche-specific edges and lines with separate errors indicate edges common for both co-occurrence networks. The nodes with back borders are common for both networks. (C)–Venn diagram showing specific and shared nodes and edges across co-occurrence networks in microbial communes from bulk and plastic-associated compost.

We generated co-occurrence networks by correlating relative abundances between microbial taxa found in ≥5% of samples from bulk and PA compost with taxa grouped at the genus level. The correlations with p-value < 0.01 were used to construct the co-occurrence networks (Fig 5A and 5B). Network from PA compost had higher proportion of positive edges (associations) compared with network from bulk compost, which suggested a greater importance of mutualistic interactions in plastic influenced environment (S6 Table). Additionally, the network from PA compost exhibited lower density, clustering coefficient and average number of neighbors compared with those from the bulk compost (S6 Table). This trend indicated a decrease in complexity of the PA compost network. Around 34% and 39% of nodes and 82% and 79% edges were unique to bulk and PA compost networks, respectively (Fig 5C and S7–S12 Tables), which suggested niche-specific nature of many potential interactions identified in the study.

At phylum level, *Firmicutes* (*Bacillaceae*, and *Thermoactinomycetaceae*) *Chytridiomycota* (*Spizellomycetaceae*) and *Zygomycota* (*Mortierella*) were the taxa unique to PA compost network (S7 Table). The associations formed by these phyla were represented by 23% of all plastic-specific association and incorporated a number of microbial partners including *Gemmatimonadetes*, *Bacteroidetes*, *Chloroflexi* and *Ascomycota* (S8 Table). *Chlamydiae* was the only phyla unique to bulk compost network (S9 Table) and was associated with *Bacteroidetes*, *Acidobacteria*, *Proteobacteria*, *Gemmatimonadetes* and the fungi *Pezizomycetes* (S10 Table).

Some structural similarities were detected between the networks from bulk and PA composts. Four major distinct clusters were formed in both networks (Figs 5A and 5B and S3). Cluster 1, containing a number of *Proteobacteria* common for both networks remained relatively intact between bulk and PA compost communities. Clusters 2 and 3 retained some similarity between the networks, but few of niche-specific nodes were found in these clusters. Detected in bulk compost network Cluster 4 was dispersed in PA compost network and a new cluster reflecting positive associations within *Firmicutes* was formed.

To further investigate the stability of the networks we looked at the correlation of the parameters of nodes and edges common to bulk and PA compost communities (S11 and S12 Tables). Co-occurrence strength (ns-score), node degree and closeness centrality had significant correlation between niches (Spearman r = 0.552 p<0.001, r = 0.521 p<0.001 and r = 0.327 p<0.05, respectively) indicating some degree of network stability. On the other hand, there was no significant correlation in node betweenness centrality between the niches (Spearman r = 0.297 p>0.05), probably because of the large number of niche-specific interaction contributing the networks.

## Discussion

The present study was carried out to assess the diversity and structure of bacterial and fungal communities in plastics contaminated compost. The structure of bacterial communities changes as the composting process progresses with the dominance of *Firmicutes* in the early stages and *Proteobacteria* in the late stages [11, 12, 35]. Our study revealed that *Proteobacteria* remained the dominant phylum across the compost facilities. *Bacteroidetes* and *Actinobacteria* were also among most of the dominant microbial taxa. These bacterial taxa were previously reported to be dominant at the latter stages in the composting process [36]. Similar to our results, *Ascomycota* was the largest fungi phylum that has been reported in compost [12, 37].

All four composting facilities studied use the windrow composting method, which produces aerobic compost. Periodic turning of the compost material (feedstock) during the composting process aerate the mass and encourage microbial activities leading to natural degradation of the organic materials to form a stable humic substance [38]. Compost recipes and feedstock is an important ingredient in the compost processing [39]. Feedstock is the main source of carbon and nitrogen for microorganism’s energy and growth and it varies from one compost facility to another. For example, carbon sources include straw, paper, woodchips and tree bark, while nitrogen sources include manure, sewage and municipal solid waste. *Ascomycota* and *Basidiomycota* are considered to be very important decomposers of hays, straws and wood in the forest floors and during composting processes [40, 41]. *Agaricomycetes* (*Basidiomycota*) and *Sordariomycete* (*Ascomycota*) were associated with hardwood degradation in compost. They have the potential to degrade lignin and cellulose in wood and other plant parts during natural decomposition [12, 41, 42]. All four compost facilities in this study used hay, straw or wood as a bulking material. This can explain why *Agaricomycetes* and *Sordariomycetes* were the dominant fungal classes in our study.

Variation in sample groupings as explained by Bray-Curtis beta-diversity showed a significant variation in community structure between compost facilities. This variation can be explained by several factors including compost chemical parameters, feedstock and age of pile. Compost chemical parameters differed between facilities and the significant correlation between some of these parameters and bacterial and fungal alpha- and beta-diversity was detected. In soils, the overall diversity, richness and evenness of bacterial community increased as the soil habitat matured [43]. Similar tendency was detected in our study, although the range of time was different in our study. The compost from Fundy compost facility was the oldest at about 10 years old and was associated with the highest alpha-diversity of bacterial community. The use of wood chips in Fundy compost facility as one of the major feedstock or bulking material can be one of the main reasons why the higher relative abandons of *Agaricomycetes* was observed in their compost compared with those from the other three facilities. However, the effect of age of the pile on the abundance of *Agaricomycetes* should not be disregarded.

We hypothesized that after prolonged storage of LDPE in compost pile i.e. between 2–10 years, the plastic could undergo some photo- and thermo-degradation and hydrolysis [44, 45], which may lead to the release of low molecular weight compounds. These compounds could be used as a carbon and nitrogen source by endogenous microorganisms in compost. It had been demonstrated that thermal and/or photolytic treatment promotes LDPE biodegradation [15]. Consequently, our hypothesis was that despite the inert nature of the plastics, microbial communities associated with partially degraded plastic would be enriched with microbial taxa either capable to utilize carbon released during abiotic plastic degradation or degrade and use the plastic for growth. For example, it was shown that after 12 weeks of composting, fungal population on the surface of polyurethanes was different from the surrounding compost community, suggesting enrichment and selection [46].

However, the results of the present study showed that there was no significant direct effect of LDPE plastic on the structure of microbial community in compost. Based on the analysis of strength and statistical significance of sample groupings, the structure of PA microbiota did not differ from microbiota from bulk compost. We also did not detect any differences in alpha-diversity and in relative abundances of microbial taxa between PA and bulk compost communities.

On the other hand, based on overall structure of co-occurrence networks, the presence of plastics affected the interaction pattern within microbial communities. We found a large number of niche-specific microbial taxa producing both cooperative and competitive association only in bulk or PA compost. Our data also indicated an increase in the importance of cooperative interactions in plastic-influenced environment compared with bulk compost. Additionally, a decrease in the complexity of the PA compost network was detected, which might be also linked to the community adaptation to plastic environment.

Schlatter *et al*. analyzed the effect of glyphosate treatment on microbial interactions in soils and showed that, despite the substantial shifts in the specific fungal interactions, the overall structure of the networks exhibited resilience to herbicide application [47]. Similarly, our data suggested a stability of overall network structure across bulk and PA compost. However, cooperation between *Firmicutes Bacillaceae* and *Thermoactinomycetaceae* was detected only in PA compost. These microorganisms were among dominant bacterial families shown to be affected by compost recipe and composting method [39]. Taking into consideration that previous studies on biodegradation of plastics in different environments identified *Bacillus* sp. as potential microorganisms involved in this process [17], our data suggest that the formation of positive associations among *Firmicutes* might not be only an adaptation response to plastic environment but also a mechanism for improvement of efficiency of plastic degradation by these bacteria.

In conclusion, our study has shown a minor effect of LDPE on diversity and structure of compost microbiome, suggesting that the presence of this type of plastics is not harmful for compost ecosystem. Our data also indicate that in some situations the analysis of network interactions might provide a better resolution for elucidation of the effects of environmental factors on microbial communities compared with the direct analysis of the communities’ diversity and structure. Likewise, it was shown that, in contrast to subtle effects of glyphosate on the structure of fungal communities, the co-occurrence network structure was robust to the herbicide application [47]. This is especially true in the situations when the minor environmental variations, like LDPE vicinity, can cause subtle impact on community structure but require a substantial shift in cooperation between community members for adaptation and probably for more efficient carbon utilization.

## Supporting information

S1 Fig Microbial taxa identified in the study. A, Bacteria, 16S rRNA gene; B, fungi, ITS.
(PPTX)

S2 Fig Principal Coordinate Analysis (PCA) of microbial communities based on bacterial 16S (A,C) and fungal ITS (B,D) community Bray-Curtis dissimilarity matrix. Each point is a different sample and the colors indicate the different sampling niches. (A,B)–Compost Facilities factor; (C,D)–Sample Type factor.
(PPTX)

S3 Fig Co-occurrence network generated by measuring abundance co-correlation between microbial taxa from bulk (A) and plastic-associated (B) compost. Correlation base network analysis showing potential interactions between bacterial and fungal genera. The size of the node is proportional to a taxon’s average relative abundance across all the samples. The lines connecting nodes (edges/associations) represent positive (blue) or negative (red) co-occurrence relationship. The solid lines indicate niche-specific edges and lines with separate errors indicate edges common for both co-occurrence networks. The nodes with back borders are common for both networks.
(PPTX)

S1 Table Description of compost facilities, age of compost and source of feedstock. (DOCX)

S2 Table Alpha-diversity of bacterial and fungal communities from plastic-associated and bulk compost. According to Tukey’s test (P < 0.05) no significant differences between each parameter were detected.
(DOCX)

S3 Table Soil chemical characteristics. For each variable, data followed by different letters are significantly different according to Tukey’s test (P < 0.05). Uppercase letters indicate significant differences between each parameter. ^a^ Cation exchange capacity.
(DOCX)

S4 Table Variation in community structure (Bray-Curtis beta-diversity distances) explained by the individual environmental variables. Adonis tests were used to assess whether beta-diversity is related to chemical soil factors, 999 permutations, R2, *p<0.05; **p<0.01, ***p<0.001. ^a^ Cation-exchange capacity
(DOCX)

S5 Table Correlation analysis considering bacterial and fungal alpha-diversity and chemical compost factors. Spearman’s rank correlation test. Significance levels are shown at *p<0.05, **p<0.01, and ***p<0.001. ^a^ Cation-exchange capacity.
(DOCX)

S6 Table Metrics for co-occurrence networks in bulk and plastic-associated compost. (DOCX)

S7 Table Nodes unique to plastic-associated compost network. The phyla unique to plastic-associated compost microbial community co-occurrence network are in bold font and in color.
(XLSX)

S8 Table Associations unique to plastic-associated compost network. The associations formed by the phyla unique to plastic-associated compost microbial community co-occurrence network are in bold font and in color.
(XLSX)

S9 Table Nodes unique to bulk compost network. The phyla unique to compost microbial community co-occurrence network are in bold font and in color.
(XLSX)

S10 Table Associations unique to bulk compost network. The associations formed by the phyla unique to bulk compost microbial community co-occurrence network are in bold font and in color.
(XLSX)

S11 Table Nodes common to both bulk and plastic-associated compost microbial community co-occurrence networks. (XLSX)

S12 Table Associations common to both bulk and plastic-associated compost microbial community co-occurrence networks. (XLSX)
