# Supplementary material for: Exploring the long-term effect of plastic on compost microbiome
Source: PLoS One. 2019 Mar 25;14(3):e0214376. doi: 10.1371/journal.pone.0214376 (PMC6433246; doi:10.1371/journal.pone.0214376)
Supplement: S1 Fig — A, Bacteria, 16S rRNA gene; B, fungi, ITS. (PPTX) [file pone.0214376.s001.pptx]

## Slide 1
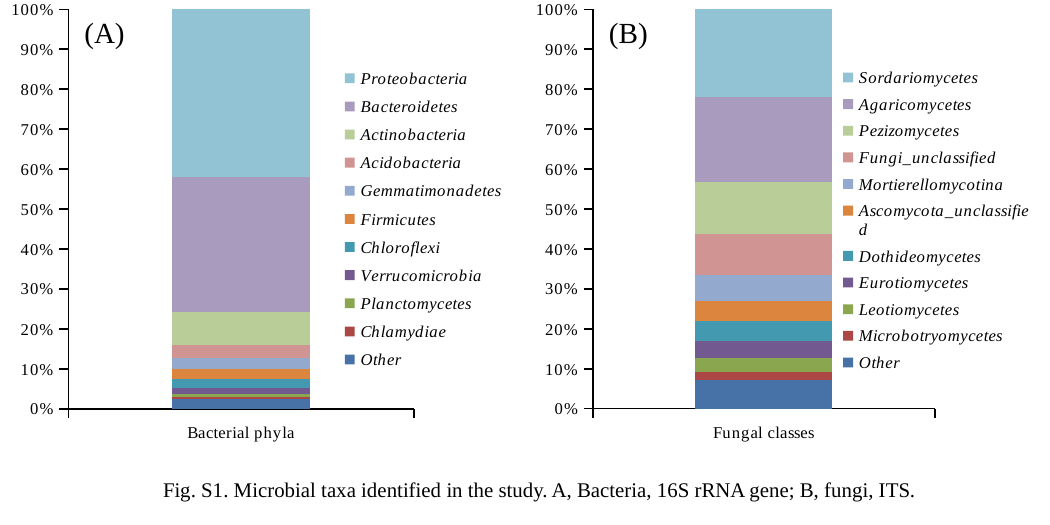

### Chart
| Category | Other | Chlamydiae | Planctomycetes | Verrucomicrobia | Chloroflexi | Firmicutes | Gemmatimonadetes | Acidobacteria | Actinobacteria | Bacteroidetes | Proteobacteria |
|---|---|---|---|---|---|---|---|---|---|---|---|
| Bacterial phyla | 0.0253516681695095 | 0.0045130748422 | 0.00747971145176 | 0.0147745716862 | 0.0212984670875 | 0.0253381424707 | 0.0275788999098 | 0.0327051397656 | 0.0831334535618 | 0.338890892696 | 0.418935978359 |
### Chart
| Category | Other | Microbotryomycetes | Leotiomycetes | Eurotiomycetes | Dothideomycetes | Ascomycota_unclassified | Mortierellomycotina | Fungi_unclassified | Pezizomycetes | Agaricomycetes | Sordariomycetes |
|---|---|---|---|---|---|---|---|---|---|---|---|
| Fungal classes | 0.0747100515463755 | 0.020006443299 | 0.0362113402062 | 0.0452963917526 | 0.0512886597938 | 0.052706185567 | 0.0667203608247 | 0.107667525773 | 0.133537371134 | 0.22181056701 | 0.227287371134 |(A)
(B)
Fig. S1. Microbial taxa identified in the study. A, Bacteria, 16S rRNA gene; B, fungi, ITS.
