# Supplementary material for: Exploring the long-term effect of plastic on compost microbiome
Source: PLoS One. 2019 Mar 25;14(3):e0214376. doi: 10.1371/journal.pone.0214376 (PMC6433246; doi:10.1371/journal.pone.0214376)
Supplement: S2 Fig — Each point is a different sample and the colors indicate the different sampling niches. (A,B)–Compost Facilities factor; (C,D)–Sample Type factor. (PPTX) [file pone.0214376.s002.pptx]

## Slide 1
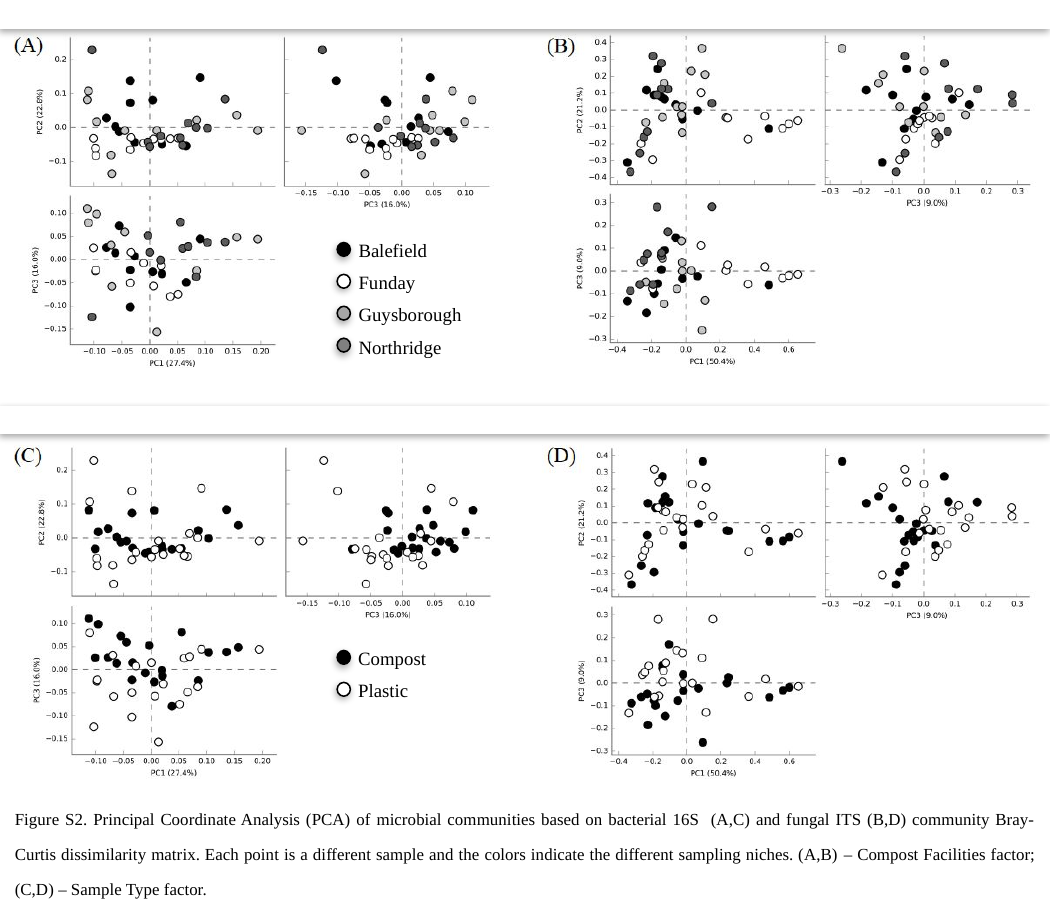

Balefield
Funday
Guysborough
Northridge
Compost
Plastic
Figure S2. Principal Coordinate Analysis (PCA) of microbial communities based on bacterial 16S (A,C) and fungal ITS (B,D) community Bray-Curtis dissimilarity matrix. Each point is a different sample and the colors indicate the different sampling niches. (A,B) – Compost Facilities factor; (C,D) – Sample Type factor.
