# Supplementary material for: Exploring the long-term effect of plastic on compost microbiome
Source: PLoS One. 2019 Mar 25;14(3):e0214376. doi: 10.1371/journal.pone.0214376 (PMC6433246; doi:10.1371/journal.pone.0214376)
Supplement: S3 Fig — Correlation base network analysis showing potential interactions between bacterial and fungal genera. The size of the node is proportional to a taxon’s average relative abundance across all the samples. The lines connecting nodes (edges/associations) represent positive (blue) or negative (red) co-occurrence relationship. The solid lines indicate niche-specific edges and lines with separate errors indicate edges common for both co-occurrence networks. The nodes with back borders are common for both networks. (PPTX) [file pone.0214376.s003.pptx]

## Slide 1
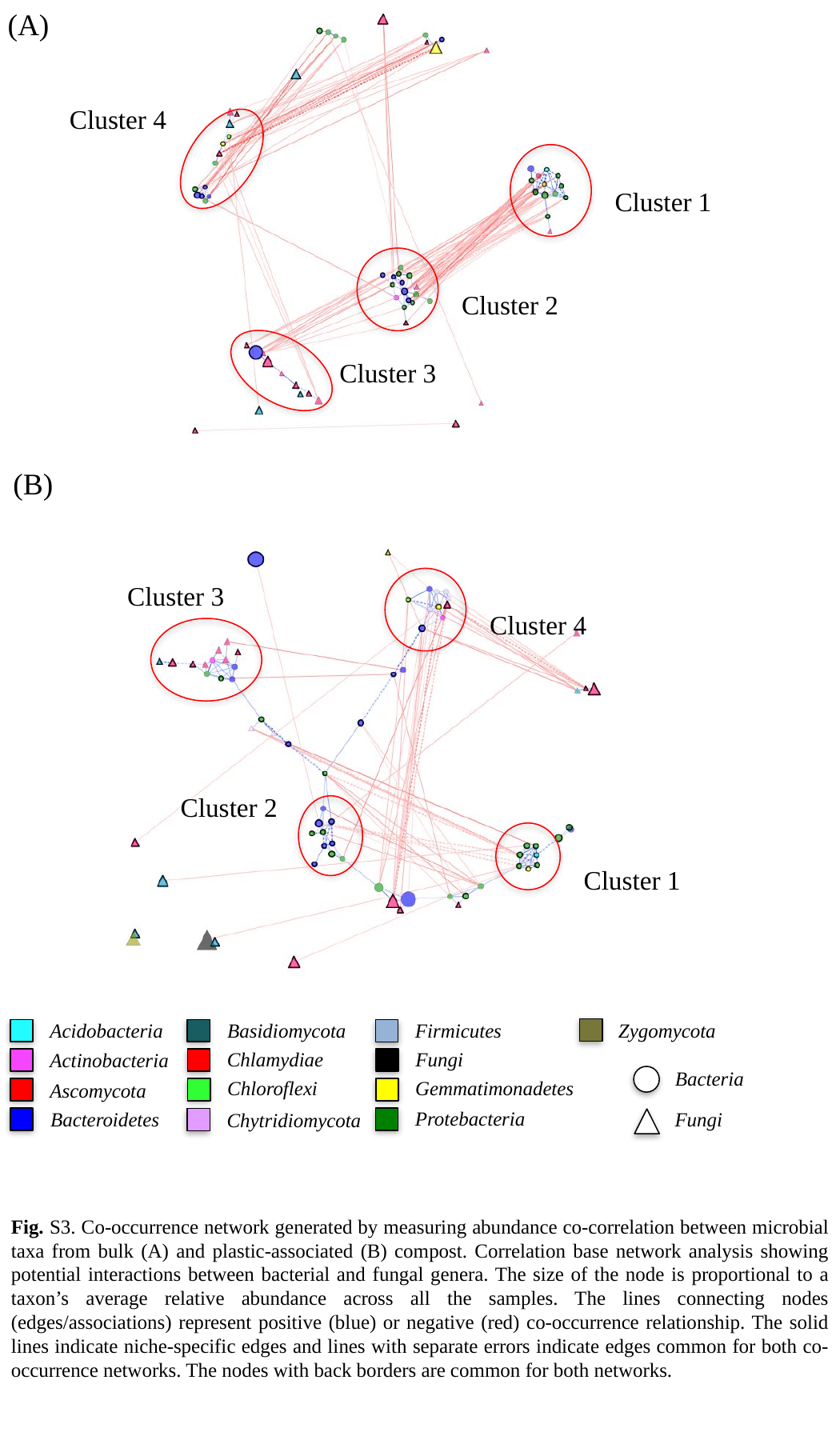

(A)
Cluster 4
Cluster 1
Cluster 2
Cluster 3
(B)
Cluster 3
Cluster 4
Cluster 2
Cluster 1
Firmicutes
Zygomycota
Acidobacteria
Basidiomycota
Fungi
Chlamydiae
Actinobacteria
Bacteria
Gemmatimonadetes
Chloroflexi
Ascomycota
Protebacteria
Fungi
Bacteroidetes
Chytridiomycota
Fig. S3. Co-occurrence network generated by measuring abundance co-correlation between microbial taxa from bulk (A) and plastic-associated (B) compost. Correlation base network analysis showing potential interactions between bacterial and fungal genera. The size of the node is proportional to a taxon’s average relative abundance across all the samples. The lines connecting nodes (edges/associations) represent positive (blue) or negative (red) co-occurrence relationship. The solid lines indicate niche-specific edges and lines with separate errors indicate edges common for both co-occurrence networks. The nodes with back borders are common for both networks.
