# Supplementary material for: Exploring the long-term effect of plastic on compost microbiome
Source: PLoS One. 2019 Mar 25;14(3):e0214376. doi: 10.1371/journal.pone.0214376 (PMC6433246; doi:10.1371/journal.pone.0214376)
Supplement: S1 Table — (DOCX) [file pone.0214376.s004.docx]

Table S1. Variation in sample groupings as explained by Bray-Curtis beta-diversity.

| Grouping | 16S (R^2^) | ITS (R^2^) |
| --- | --- | --- |
| Facility^a^ | 0.264*** | 0.277*** |
| Age of Pile | 0.105*** | 0.094*** |
| Sample Type^c^ | 0.026 | 0.028 |
| Sample type (Balefill)^d^ | 0.026 | 0.028 |
| Sample type (Guysborough) | 0.026 | 0.028 |
| Sample type (Fundy) | 0.026 | 0.028 |
| Sample type (Northridge) | 0.026 | 0.028 |

Adonis tests were used to assess whether beta-diversity is related to sample groupings, 999 permutations, R^2^, ***P < 0.001.

^a^ Bulk and plastic associated microbial communities from each compost-processing facility combined.

^b^ Bulk and plastic associated microbial communities from the piles with the same age combined.

^c^ Bulk compost vs. plastic associated microbial communities from all compost-processing facilities combined.

^d^ Bulk compost vs. plastic associated microbial communities from individual faculty.
