# Supplementary material for: Exploring the long-term effect of plastic on compost microbiome
Source: PLoS One. 2019 Mar 25;14(3):e0214376. doi: 10.1371/journal.pone.0214376 (PMC6433246; doi:10.1371/journal.pone.0214376)
Supplement: S2 Table — According to Tukey’s test (P < 0.05) no significant differences between each parameter were detected. (DOCX) [file pone.0214376.s005.docx]

Table S2. Alpha-diversity of bacterial and fungal communities from plastic-associated and bulk compost

| Sample type/  Metrics | Chao1 Richness | | Simpson Evenness | | Shannon Diversity | |
| --- | --- | --- | --- | --- | --- | --- |
|  | 16S | ITS | 16S | ITS | 16S | ITS |
| Bulk compost | 1568 | 104 | 0.154 | 0.160 | 8.651 | 4.151 |
| Plastic-associated compost | 1410 | 96 | 0.152 | 0.125 | 8.373 | 3.870 |

According to Tukey’s test (P < 0.05) no significant differences between each parameter were detected.
