# Supplementary material for: Exploring the long-term effect of plastic on compost microbiome
Source: PLoS One. 2019 Mar 25;14(3):e0214376. doi: 10.1371/journal.pone.0214376 (PMC6433246; doi:10.1371/journal.pone.0214376)
Supplement: S3 Table — For each variable, data followed by different letters are significantly different according to Tukey’s test (P < 0.05). Uppercase letters indicate significant differences between each parameter. a Cation exchange capacity. (DOCX) [file pone.0214376.s006.docx]

Table S3. Soil chemical characteristics

| Compost facilities/Soil parameters | Balefill | Guysborough | Fundy | Northridge |
| --- | --- | --- | --- | --- |
| Nitrogen (%) | 1.636^B^ | 3.456^A^ | 1.664^B^ | 1.782^B^ |
| pH (pH units) | 6.98^C^ | 7.42^AB^ | 7.13^BC^ | 7.68^A^ |
| Organic Matter (%) | 15.9^B^ | 29.2^A^ | 19.7^B^ | 18.0^B^ |
| P2O5 (kg/ha) | 2659.3^AB^ | 2556.8^AB^ | 2142.8^B^ | 3050.3^A^ |
| K_2_O (kg/ha) | 2417.3^AB^ | 4439.0^A^ | 2519.0^B^ | 2831.3^AB^ |
| Calcium (kg/ha) | 9766.3^BC^ | 11178.8^B^ | 8179.6^C^ | 16431.3^A^ |
| Magnesium (kg/ha) | 1195.3^AB^ | 1111.2^BC^ | 936.4^C^ | 1408.5^A^ |
| Sodium (kg/ha) | 548.0^A^ | 1848.2^A^ | 850.8^A^ | 1237.8^A^ |
| Sulfur (kg/ha) | 429.0^A^ | 176.2^B^ | 165.4^B^ | 143.0^B^ |
| Aluminum (ppm) | 121.3A^B^ | 82.2^B^ | 222.0^A^ | 66.75^B^ |
| Boron (ppm) | 2.557^A^ | 3.054^A^ | 2.594^A^ | 3.382^A^ |
| Copper (ppm) | 2.033^A^ | 1.684^A^ | 2.492^A^ | 2.485^A^ |
| Iron (ppm) | 206.3^AB^ | 150.0^B^ | 253.6^A^ | 173.5^B^ |
| Manganese (ppm) | 124.3^A^ | 47.6^B^ | 44.2^B^ | 59.5^B^ |
| Zinc (ppm) | 41.21^A^ | 36.84^A^ | 27.36^A^ | 24.73^A^ |
| CEC^a^ (meq/100 g) | 33.4^BC^ | 41.4^B^ | 29.42^C^ | 52.7^A^ |

For each variable, data followed by different letters are significantly different according to Tukey’s test (P < 0.05). Uppercase letters indicate significant differences between each parameter.

^a^ Cation exchange capacity.
