# Supplementary material for: Exploring the long-term effect of plastic on compost microbiome
Source: PLoS One. 2019 Mar 25;14(3):e0214376. doi: 10.1371/journal.pone.0214376 (PMC6433246; doi:10.1371/journal.pone.0214376)
Supplement: S4 Table — Adonis tests were used to assess whether beta-diversity is related to chemical soil factors, 999 permutations, R2, *p<0.05; **p<0.01, ***p<0.001. a Cation-exchange capacity (DOCX) [file pone.0214376.s007.docx]

Table S4. Variation in community structure (Bray-Curtis beta-diversity distances) explained by the individual environmental variables

| Amplicon/Soil parameters | 16S | ITS |
| --- | --- | --- |
| Nitrogen | 0.427** | 0.305** |
| pH | 0.172* | 0.304** |
| Organic Matter | 0.377** | 0.182* |
| P_2_O_5_ | -0.027 | 0.076 |
| K_2_O | 0.539*** | 0.142 |
| Calcium | 0.077 | 0.203* |
| Magnesium | -0.009 | 0.091 |
| Sodium | 0.489*** | 0.095 |
| Sulfur | 0.167* | 0.0543 |
| Aluminum | 0.247** | 0.195* |
| Boron | 0.137 | -0.030 |
| Copper | 0.126 | 0.009 |
| Iron | 0.518*** | 0.277** |
| Manganese | 0.154 | 0.070 |
| Zinc | -0.043 | 0.048 |
| CEC^a^ | 0.243* | 0.225* |

Adonis tests were used to assess whether beta-diversity is related to chemical soil factors, 999 permutations, R2, *p<0.05; **p<0.01, ***p<0.001.

^a^ Cation-exchange capacity
