# Supplementary material for: Exploring the long-term effect of plastic on compost microbiome
Source: PLoS One. 2019 Mar 25;14(3):e0214376. doi: 10.1371/journal.pone.0214376 (PMC6433246; doi:10.1371/journal.pone.0214376)
Supplement: S5 Table — Spearman’s rank correlation test. Significance levels are shown at *p<0.05, **p<0.01, and ***p<0.001. a Cation-exchange capacity. (DOCX) [file pone.0214376.s008.docx]

Table S5. Correlation analysis considering bacterial and fungal alpha-diversity and chemical compost factors

| Compost factors/  alpha-diversity parameters | Shannon diversity | |
| --- | --- | --- |
|  | 16S | ITS |
| pH | -0.331 | 0.212 |
| Nitrogen | -0.342 | -0.346 |
| OM | -0.114 | -0.469 |
| P_2_O_5_ | -0.505* | -0.218 |
| K_2_O | -0.341 | -0.321 |
| Ca | -0.556* | -0.189 |
| Mg | -0.346 | 0.164 |
| Na | -0.289 | -0.304 |
| S | -0.299 | -0.297 |
| Al | 0.743*** | 0.380 |
| Co | 0.064 | 0.346 |
| Fe | 0.689** | 0.069 |
| Mn | -0.433 | 0.409 |
| Zn | -0.010 | 0.490 |
| CEC^a^ | -0.532* | -0.340 |

Spearman’s rank correlation test. Significance levels are shown at *p<0.05, **p<0.01, and ***p<0.001. ^a^ Cation-exchange capacity.
