# Supplementary material for: Exploring the long-term effect of plastic on compost microbiome
Source: PLoS One. 2019 Mar 25;14(3):e0214376. doi: 10.1371/journal.pone.0214376 (PMC6433246; doi:10.1371/journal.pone.0214376)
Supplement: S6 Table — (DOCX) [file pone.0214376.s009.docx]

Table S6. Metrics for co-occurrence networks in bulk and plastic-associated compost.

|  | Plastic | Bulk |
| --- | --- | --- |
| Number of nodes | 72 | 67 |
| Number of edges | 166 | 204 |
| Positive interactions | 115 | 97 |
| Negative interactions | 51 | 107 |
| Positive/Negative | 2.255 | 0.906 |
| Network density | 0.065 | 0.092 |
| Clustering coefficient | 0.386 | 0.426 |
| Avg. number of neighbors | 4.611 | 6.090 |
